# Persistence of *Helicobacter pylori* Infection Despite Therapy: Eight-Year Real-World Experience from a Saudi Tertiary Center

**DOI:** 10.3390/jcm15083106

**Published:** 2026-04-18

**Authors:** Yara Alassaf, Sadeem Aleid, Ftoon Alenezi, Ghadah Alhabs, Taif Almutairi, Seham Alsalamah, Nouf Althabit, Somaiya Alshabeer, Abdulellah Almohaya, Mohamed Albabtain, Mohammad Bosaeed

**Affiliations:** 1 College of Medicine, King Saud Bin Abdulaziz University for Health Sciences, Riyadh 11481, Saudi Arabiaftoon.e.alenezi@hotmail.com (F.A.); ghadahalhebs@gmail.com (G.A.); taifsalehh2@gmail.com (T.A.); n.althabit@hotmail.com (N.A.); almohayaab1@mngha.med.sa (A.A.); 2 King Abdulaziz Medical City, Ministry of National Guard Health Affairs, Riyadh 11426, Saudi Arabia; somaiyah.alshabeer@gmail.com (S.A.); albabtainm01@mngha.med.sa (M.A.); 3 King Abdullah International Medical Research Center, Riyadh 11481, Saudi Arabia

**Keywords:** *Helicobacter pylori*, eradication failure, antimicrobial resistance, treatment outcome, Saudi Arabia

## Abstract

**Background/Objectives**: Rising antimicrobial resistance threatens *Helicobacter pylori* eradication worldwide, yet real-world data on microbiologically confirmed treatment outcomes from the Middle East remain scarce. This study aimed to determine the confirmed eradication failure rate among treatment-naïve patients, identify independent predictors of failure, and characterize gaps in post-treatment testing practices. **Methods**: This retrospective cohort study included treatment-naïve adults diagnosed with *H. pylori* infection at King Abdulaziz Medical City in Riyadh between January 2015 and August 2023. The primary outcome was microbiologically confirmed eradication failure, defined exclusively as a positive post-treatment test of cure (urea breath test, stool antigen, or endoscopic biopsy). Exploratory logistic regression was used to identify factors associated with treatment failure. **Results**: Of 850 patients, 196 (23.1%) had a documented post-treatment eradication test. The confirmed failure rate was 33.7% (66/196). In multivariate analysis, atrophic gastritis (aOR 3.22, 95% CI: 1.29–8.02; *p* = 0.012) and pregnancy (aOR 12.76, 95% CI: 1.79–91.06; *p* = 0.011) were independently associated with failure. No treatment regimen was significantly associated with eradication outcome. **Conclusions**: One in three tested patients failed first-line eradication, and over three-quarters of treated patients never received a confirmatory test of cure. These findings support transitioning to bismuth-based quadruple therapy in line with current guidelines and local resistance data, mandating routine post-treatment eradication testing, and establishing antimicrobial susceptibility surveillance.

## 1. Introduction

*Helicobacter pylori* infection (HPI) is the leading cause of infection-associated cancer globally and remains one of the most prevalent bacterial infections worldwide [1,2,3,4]. In Saudi Arabia, the reported prevalence appears to be declining, from >70% seropositivity in the 1990s to 10–32% in more recent studies [5,6,7,8,9]. Globally, systematic reviews estimate that 44–50% of the world population harbors *H. pylori*, with higher rates in developing regions [9,10].

Current guidelines recommend several first-line regimens, including clarithromycin-based triple therapy, bismuth quadruple therapy, and concomitant therapy [11,12,13]. However, rising clarithromycin resistance—approaching 40% of isolates in Saudi Arabia, with nearly one-third identified in treatment-naïve patients—threatens the efficacy of traditional triple therapy [14]. The American College of Gastroenterology (ACG) 2024 and local 2022 guidelines now favor bismuth-based quadruple therapy in regions where clarithromycin resistance exceeds 15% [11,13]. Achieving eradication on the first attempt is critical to minimizing additional treatment courses, costs, and the selection of resistant strains [15,16].

Eradication failure is multifactorial, with antibiotic resistance as the principal contributor. Other factors include treatment duration, adherence, gastric pathology, host genetic polymorphisms (CYP2C19), and bacterial virulence factors [17,18,19,20,21,22]. Despite the clinical relevance of these factors, data from Saudi Arabia on eradication outcomes in treatment-naïve populations remain limited, with few studies reporting confirmed microbiological failure rates or evaluating predictors of persistence [15,23].

This study presents a real-world cohort of treatment-naïve patients with HPI in Saudi Arabia. We aimed to evaluate confirmed eradication outcomes, characterize treatment patterns, and identify clinical factors linked to microbiologically confirmed treatment failure, providing evidence to guide local clinical practice and updates to guidelines.

## 2. Materials and Methods

### 2.1. Study Method, Settings, and Participants

This is a retrospective cohort study of patients newly diagnosed with *H. pylori* infection between January 2015 and August 2023 at King Abdulaziz Medical City in Riyadh (KAMC-RD), a tertiary care center under the Ministry of National Guard Health Affairs. All eligible adult patients (≥18 years) during the study period were included using consecutive sampling. Inclusion criteria required a treatment-naïve confirmed diagnosis of *H. pylori* based on a positive urea breath test (UBT), stool antigen test (enzyme immunoassay), or endoscopic biopsy. Patients treated empirically without confirmed testing were excluded.

### 2.2. Treatment and Follow-Up

Treatment regimens were prescribed at physician discretion and included clarithromycin-based triple therapy (PPI + amoxicillin + clarithromycin [PAC]), metronidazole-based triple therapy (PPI + metronidazole + clarithromycin [PMC] or PPI + amoxicillin + metronidazole [PAM]), concomitant therapy (PPI + amoxicillin + clarithromycin + metronidazole [PACM]), bismuth quadruple therapy (PPI + bismuth + metronidazole + tetracycline [PMBT]), and levofloxacin-based therapy (PPI + amoxicillin + levofloxacin [PAL]). Treatment duration was extracted from medication initiation and termination dates, where documented. The institutional protocol recommends a test of cure (UBT) performed after a minimum 4-week washout period for antibiotics and bismuth, and a 2-week washout for proton pump inhibitors.

### 2.3. Outcome Definitions

Eradication failure (the primary outcome) was defined exclusively as a positive post-treatment test of cure (UBT, stool antigen, or endoscopy-based test) following completion of the treatment course. Symptom persistence alone was not considered evidence of eradication failure. Patients without a documented post-treatment eradication test were classified as “not assessable” for the primary analysis.

Documented treatment completion was defined as chart documentation of ≥80% of the prescribed regimen completed without interruption. This is a proxy measure based on retrospective chart review and does not represent a validated adherence assessment.

### 2.4. Data Collection

Data were collected from the hospital’s electronic medical records (BESTCare 2.0) and included demographics (age, sex, BMI, social and smoking status), diagnosis details (date, diagnostic method), comorbidities (diabetes, hypertension, dyslipidemia, atrophic gastritis, peptic ulcer disease, pregnancy, malignancy, IBS, GERD), presenting symptoms, treatment information (medications, duration, documented treatment completion), eradication status after each treatment course, and post-treatment complications at six months (cardiovascular events, anemia, liver conditions, dyslipidemia).

### 2.5. Statistical Analysis

Categorical data were presented as frequencies and percentages; numerical data as median and interquartile range or mean and standard deviation as appropriate. Comparisons used the chi-squared or Fisher’s exact test for categorical variables and the Mann–Whitney U test for continuous variables. Univariate and multivariate logistic regression were performed to identify factors associated with confirmed eradication failure. Firth’s penalized logistic regression was used to handle sparse data (pregnancy: n = 7, 6 failures). Variables with *p* < 0.20 in univariate analysis were included in the multivariate model (BMI, sex, hypertension, atrophic gastritis, documented treatment completion, pregnancy). Smoking was excluded from the multivariate model due to perfect collinearity with sex in the tested subgroup. A separate regression analysis assessed the association between treatment regimen and eradication failure, with PAC as the reference category. This analysis is exploratory and associative; the observational design with physician-directed treatment allocation precludes causal interpretation. A *p*-value ≤0.05 was considered statistically significant.

## 3. Results

### 3.1. Study Population

A total of 850 patients met the inclusion criteria. Mean age was 39.1 ± 14.6 years (median 36, IQR 27–49), with a female predominance (573, 67.4%) and a mean BMI of 28.3 ± 6.8 kg/m^2^. Among comorbidities, dyslipidemia was the most frequent (303, 35.6%), followed by diabetes mellitus (146, 17.2%; type 2 in 143), hypertension (139, 16.4%), atrophic gastritis (110, 12.9%), and irritable bowel syndrome (105, 12.4%). Smoking was reported in 75 (8.8%). The most common presenting symptoms were abdominal pain (566, 66.6%), dyspepsia (332, 39.1%), heartburn (219, 25.8%), and nausea (163, 19.2%). Diagnosis was by UBT in 756 (88.9%), endoscopic biopsy in 141 (16.6%), and stool antigen assay in 30 (3.5%) (Table 1 and Table 2).

### 3.2. Treatment Regimens:

All patients received a proton pump inhibitor. First-line triple therapy was administered to 777 patients (91.4%), with clarithromycin-based PAC being the most common (661, 77.8%). Metronidazole, a nitroimidazole, was used in 173 patients (20.4%) during the first course across PMC (n = 26), PAM, and PACM (n = 54) regimens. Bismuth-containing quadruple therapy was prescribed in only 2 patients as first-line, and levofloxacin-based therapy in 1 patient. Documented treatment completion was recorded in 513 (60.4%) patients. Second and third courses were required in 176 (20.7%) and 65 (7.6%) patients, respectively (Table 3 and Table S1).

### 3.3. Treatment Duration

Antibiotic duration data (PPI excluded) were available for 379/850 patients (44.6%). The median duration was 14 days (IQR 13–14). Among the 57 test-confirmed patients with complete antibiotic data, no significant association was found between duration and eradication failure (OR per day: 0.997, 95% CI: 0.987–1.006, *p* = 0.491; ≤14 vs. >14 days: OR 3.22, 95% CI: 0.606–17.112, *p* = 0.170), (Table S4).

### 3.4. Eradication Assessment and Primary Outcome

Of 850 patients, 196 (23.1%) had a documented post-treatment eradication test (UBT: 174; stool antigen: 16; endoscopy: 6) and constituted the primary analysis population. The remaining 648 (76.2%) lacked a documented test of cure, with 6 (0.7%) missing. Among the 196 tested patients, the confirmed eradication failure rate was 33.7% (66/196; 95% CI: 27.4–40.5%). Eradication success was 66.3% (130/196). After the second course (N = 176), eradication success was 51.1% (90/176). After the third course (N = 65), success was 49.2% (32/65). Persistent symptoms were documented in 138/850 (16.2%) using the original composite definition (Table 3).

### 3.5. Factors Associated with Eradication Failure:

In univariate Firth’s penalized logistic regression (N = 196), BMI per unit (OR 1.03, 95% CI: 1.00–1.07; *p* = 0.035), atrophic gastritis (OR 3.67, 95% CI: 1.61–8.40; *p* = 0.002), and pregnancy (OR 9.28, 95% CI: 1.36–63.43; *p* = 0.023) were significantly associated with eradication failure. Hypertension showed a non-significant trend toward protection (OR 0.27, 95% CI: 0.07–1.09; *p* = 0.066). Abdominal pain (OR 1.06, *p* = 0.857), dyspepsia (OR 0.94, *p* = 0.848), and clarithromycin-based triple therapy (included in the regimen-specific model) were not associated with failure (Table 4).

In the multivariate model (n = 191 complete cases), atrophic gastritis (aOR 3.22, 95% CI: 1.29–8.02; *p* = 0.012) and pregnancy (aOR 12.76, 95% CI: 1.79–91.06; *p* = 0.011) remained independently associated with failure. BMI showed a non-significant trend (aOR 1.04, 95% CI: 0.99–1.08; *p* = 0.128). In a separate regimen-specific analysis using PAC as the reference, no regimen was significantly associated with eradication outcome (PACM aOR 0.92, *p* = 0.906; PMC aOR 1.63, *p* = 0.668; Other aOR 0.85, *p* = 0.752) (Table 4 and Table S3).

### 3.6. Complications

Among the well-documented complications of *H. pylori* infection in the full cohort (N = 850), peptic ulcer disease was present in 10 patients (1.2%), chronic atrophic gastritis in 110 (12.9%), MALT lymphoma in 2 (0.2%), and idiopathic thrombocytopenic purpura in 1 (0.1%). During post-treatment follow-up, no cases of gastric cancer were observed. Regarding metabolic and cardiovascular complications after treatment, dyslipidemia was noted in 28 (3.3%), ischemic heart disease in 3 (0.4%), myocardial infarction in 1 (0.1%), and atherosclerosis in 1 (0.1%). The low event rates prevent meaningful statistical comparisons between eradication success and failure groups (Table S2).

## 4. Discussion

This study provides a real-world assessment of *H. pylori* eradication outcomes among treatment-naïve patients in Saudi Arabia. The confirmed eradication failure rate of 33.7% among the 196 patients with documented test-of-cure results substantially exceeds internationally accepted thresholds. Atrophic gastritis and pregnancy were the only independent predictors of failure. Critically, 76.2% of patients lacked a post-treatment eradication test, revealing a fundamental gap in clinical practice [24,25].

Several factors likely contribute to the high failure rate observed. First, clarithromycin-based triple therapy (PAC), used in 77.8% of first-line prescriptions, may be insufficient given local clarithromycin resistance rates approaching 40% [14]. At this resistance level, predicted eradication rates with standard triple therapy drop below 70%, consistent with our findings [11]. Second, atrophic gastritis—present in 25.8% of failures versus 8.5% of successes—was the strongest predictor of failure (aOR 3.22), likely reflecting reduced antibiotic effectiveness due to altered gastric pH and mucosal architecture [26,27,28]. Third, antibiotic duration data, available for only 44.6% of patients, showed a median of 14 days, though the limited overlap with the tested subgroup (n = 57) prevented a robust duration–outcome analysis. Finally, the absence of antimicrobial susceptibility testing means that all therapy in this cohort was empirically prescribed without knowledge of individual resistance profiles.

In the regimen-specific analysis, no treatment regimen was significantly associated with eradication compared to clarithromycin triple therapy (Table S3). However, the observational design with physician-guided regimen choice prevents meaningful comparison of treatment effectiveness, as different regimens were prescribed to clinically distinct populations. The high overall failure rate (33.7%) in a cohort mainly treated with PAC (77.8%), along with published local clarithromycin resistance rates approaching 40%, offers indirect evidence that empirical clarithromycin-based triple therapy may no longer be sufficient as the standard first-line treatment in this setting. This aligns with the ACG 2024 and Maastricht VI guidelines, which recommend bismuth quadruple therapy where clarithromycin resistance exceeds 15% [11,13].

Based on our findings and published resistance data, we propose the following considerations for clinical practice: (1) Bismuth quadruple therapy should be considered the preferred first-line treatment in settings where clarithromycin resistance exceeds 15%, in line with the ACG 2024 recommendation [11]. (2) Treatment duration should be standardized to 14 days, supported by strong evidence from randomized trials and current guidelines. (3) Routine post-treatment test-of-cure should be mandated—the finding that 76.2% of patients lacked eradication confirmation highlights a significant quality gap that hampers treatment effectiveness and leads to unreliable outcomes. (4) Antibiotic stewardship principles should guide prescribing, including documentation of treatment duration, regimen rationale, and follow-up testing as standard practice across institutions.

The absence of culture and antimicrobial susceptibility testing in our cohort is a significant limitation, highlighting a broader gap in regional practice. Susceptibility-guided therapy should be strongly considered for: (1) patients who fail first-line therapy, particularly before starting a third course; (2) patients with penicillin allergy, where treatment options are limited; and (3) populations with high resistance prevalence and uncertain empirical regimen choices. Establishing a national or institutional *H. pylori* susceptibility surveillance program would provide the resistance data needed to guide empirical therapy and track resistance trends over time.

This study has several key limitations. First, its retrospective design introduces selection bias due to single-center recruitment, information bias from chart-based outcome collection, and confounding by indication because treatment regimens were physician-selected rather than randomized. Second, only 23.1% of patients had a documented post-treatment eradication test, greatly limiting statistical power; the 76.2% who were untested may differ systematically from those tested. Third, documented treatment completion is a chart-review-based proxy that likely overestimates true adherence; it does not reflect validated adherence assessments and should not be used to imply causality [29]. Fourth, the lack of antimicrobial susceptibility testing prevents resistance-adjusted analysis. Fifth, data on prior antibiotic and macrolide exposure were not available, preventing adjustment for this important confounder. Sixth, antibiotic duration data were available for only 44.6% of patients, limiting the analysis of duration and outcomes. Seventh, smoking status was perfectly collinear with sex in the tested subgroup and could not be included in the multivariate model. The strengths of this study include its large initial sample size, comprehensive data collected from an advanced EMR system (BESTCare 2.0), the use of Firth’s penalized regression to properly manage sparse outcomes, and the methodological adjustment of the primary outcome to microbiologically confirmed failure—although this reduces the assessable population, it significantly enhances the validity of the reported eradication rate and predictor analysis.

## 5. Conclusions

In this retrospective cohort of treatment-naïve patients, one in three patients with a documented test of cure failed first-line *H. pylori* eradication, with atrophic gastritis and pregnancy identified as independent associated factors. No treatment regimen was significantly associated with eradication outcome. Perhaps equally concerning, over three-quarters of treated patients never received confirmatory post-treatment testing—a fundamental practice gap that undermines both individual patient management and institutional quality assessment.

These findings, interpreted alongside current guideline recommendations and local clarithromycin resistance data (~40%), support four actionable priorities: (1) adopting bismuth-based quadruple therapy as the preferred first-line regimen; (2) mandating routine post-treatment test-of-cure as standard of care; (3) standardizing 14-day treatment duration; and (4) establishing antimicrobial susceptibility surveillance to inform empirical therapy selection. Prospective, multicenter studies with susceptibility-guided treatment arms are needed to validate these findings and optimize *H. pylori* management in the region.

## Figures and Tables

**Table 1:** Baseline Characteristics of Patients with *H. pylori* Infection (N = 850).

Variable		N (%) or Mean ± SD
Demographics		
Age, Years, Mean ± Sd		39.1 ± 14.6
Age, Years, Median (Iqr)		36 (27–49)
Sex		
	Male	277 (32.6)
	Female	573 (67.4)
Weight (Kg), Mean ± Sd		73.6 ± 18.4
Height (Cm), Mean ± Sd		172.2 ± 338.0
BMI (Kg/M^2^), Mean ± Sd		28.3 ± 6.8
Smoking		
	Yes	75 (8.8)
	No	619 (72.8)
	Missing	156 (18.4)
Concurrent Illness		
	Dyslipidemia	303 (35.6)
	Hypertension	139 (16.4)
	Diabetes mellitus	146 (17.2)
	Type 1	3 (0.4)
	Type 2	143 (16.8)
	Atrophic Gastritis	110 (12.9)
	IBS	105 (12.4)
	GERD	92 (10.8)
	Heart Failure	45 (5.3)
	Pregnancy	41 (4.8)
	Malignancy	19 (2.2)
	Peptic Ulcer Disease	10 (1.2)
	MALT Lymphoma	2 (0.2)
	Crohn’s Disease	1 (0.1)
	Ulcerative Colitis	1 (0.1)

**Table 2:** Clinical Presentation and Diagnostic Testing.

Variable	N (%)
Presenting Symptoms	
Abdominal Pain	566 (66.6)
Dyspepsia	332 (39.1)
Heartburn	219 (25.8)
Nausea	163 (19.2)
Bloating	82 (9.6)
Vomiting	81 (9.5)
Anemia	58 (6.8)
Diarrhea	57 (6.7)
Weight Loss	40 (4.7)
Loss of Appetite	39 (4.6)
Melena	22 (2.6)
Hematemesis	9 (1.1)
Halitosis	5 (0.6)
Diagnostic Testing	
Urea Breath Test	756 (88.9)
Biopsy	141 (16.6)
Stool Antigen Assay	30 (3.5)

**Table 3:** First-Line Treatment Regimens (N = 850).

Regimen	N (%)	Adherence	Failure *
Triple Therapy	777 (91.4)		
PAC (Clarithromycin-based)	661 (77.8)	404 (61.1)	52/151 (34.4)
PMC (Metronidazole-based)	26 (3.1)	21 (80.8)	1/4 (25.0)
PAL (Levofloxacin-based)	1 (0.1)	1 (100)	0/0
Other Triple	89 (10.5)		
Quadruple Therapy	61 (7.2)		
Non-Bismuth (PACM)	54 (6.4)	41 (75.9)	4/13 (30.8)
Bismuth (PMBT)	2 (0.2)	2 (100)	0/0
Other Quadruple	5 (0.6)		
Others	12 (1.4)		

* Eradication failure: confirmed positive post-treatment test only (tested subgroup). Adherence = documented treatment completion (≥80%).

**Table 4:** Exploratory Associative Analysis: Factors Associated with Confirmed Eradication Failure After First Course (Test-Confirmed, N = 196).

Variable	OR (95% CI)	*p*	aOR (95% CI)	*p*
	Univariate		Multivariate	
Age (per year)	0.99 (0.97–1.01)	0.317		
Sex (Male)	1.71 (0.89–3.26)	0.105	1.12 (0.44–2.83)	0.815
BMI (per unit)	1.03 (1.00–1.07)	0.035 *	1.04 (0.99–1.08)	0.128
Smoking ‡	1.71 (0.89–3.26)	0.105		
Diabetes mellitus	1.22 (0.61–2.42)	0.577		
Hypertension	0.27 (0.07–1.09)	0.066	0.47 (0.11–1.97)	0.301
Dyslipidemia	0.95 (0.52–1.74)	0.865		
GERD	1.44 (0.58–3.56)	0.432		
Atrophic Gastritis	3.67 (1.61–8.40)	0.002 *	3.22 (1.29–8.02)	0.012 *
Macroscopic Lesions	1.54 (0.76–3.12)	0.234		
Documented treatment completion	1.65 (0.88–3.08)	0.115	1.28 (0.64–2.57)	0.484
Pregnancy	9.28 (1.36–63.43)	0.023 *	12.76 (1.79–91.06)	0.011 *

* *p* < 0.05. ‡ Smoking excluded from multivariate model due to perfect collinearity with sex (r = 1.0).

## Data Availability

The data presented in this study are available on request from the corresponding author.

## Supplementary Materials

The following supporting information can be downloaded at: https://www.mdpi.com/article/10.3390/jcm15083106/s1, Table S1: Treatment Regimen Distribution by Course; Table S2: Risk Factors for Treatment Failure and Post-Treatment Complications (N = 850); Table S3: Association Between Treatment Regimen and Eradication Failure (N = 196); Table S4: Antibiotic Therapy Duration and Association with Eradication Outcome.

## Abbreviations

The following abbreviations are used in this manuscript:

HPI	*Helicobacter pylori* infection
*H. pylori*	*Helicobacter pylori*
UBT	Urea breath test
PPI	Proton pump inhibitor
PMC	PPI + Metronidazole + Clarithromycin
PACM	PPI + Amoxicillin + Clarithromycin + Metronidazole
GERD	Gastroesophageal reflux disease
IBD	Inflammatory Bowel Disease
MALT	Mucosa-associated lymphoid tissue
SAT	Stool antigen
