# Supplementary material for: Persistence of Helicobacter pylori Infection Despite Therapy: Eight-Year Real-World Experience from a Saudi Tertiary Center
Source: J Clin Med. 2026 Apr 18;15(8):3106. doi: 10.3390/jcm15083106 (PMC13117063; doi:10.3390/jcm15083106)
Supplement: Supplementary file 1 [file jcm-15-03106-s001.zip › jcm-4183012-supplementary.pdf]

Supplementary Material

Table S1. Treatment Regimen Distribution by Course.

**First Course (N = 850)**				
**Regimen**	**N (%)**	**Adherent****	**Non-adh.**	**Failure***
PAC	661 (77.8)	404 (61.1)	252 (38.1)	52/151 (34.4)
PACM	54 (6.4)	41 (75.9)	12 (22.2)	4/13 (30.8)
PMC	26 (3.1)	21 (80.8)	5 (19.2)	1/4 (25.0)
PMBT	2 (0.2)	2 (100)	0 (0)	0/0 (—)
PAL	1 (0.1)	1 (100)	0 (0)	0/0 (—)
Other	106 (12.5)	44 (41.5)	60 (56.6)	9/28 (32.1)
**Second Course (N = 176)**				
PAC	106 (60.2)	67 (63.2)	36 (34.0)	52 (49.1)
PACM	19 (10.8)	9 (47.4)	10 (52.6)	12 (63.2)
PMC	8 (4.5)	2 (25.0)	5 (62.5)	4 (50.0)
PAL	7 (4.0)	7 (100)	0 (0)	2 (28.6)
PMBT	1 (0.6)	1 (100)	0 (0)	1 (100)
Other	35 (19.9)	13 (37.1)	20 (57.1)	15 (42.9)
**Third Course (N = 65)**				
PAC	30 (46.2)	25 (83.3)	5 (16.7)	16 (53.3)
PACM	11 (16.9)	6 (54.5)	5 (45.5)	5 (45.5)
PAL	3 (4.6)	2 (66.7)	1 (33.3)	2 (66.7)
PMC	2 (3.1)	1 (50.0)	1 (50.0)	2 (100)
PMBT	2 (3.1)	2 (100)	0 (0)	1 (50.0)
Other	17 (26.2)	10 (58.8)	7 (41.2)	7 (41.2)

** 1st course failure: confirmed positive test only. 2nd/3rd course: uses available eradication data. PAC = PPI + Amoxicillin + Clarithromycin; PMC = PPI + Metronidazole + Clarithromycin; PAL = PPI + Amoxicillin + Levofloxacin; PACM = PAC + Metronidazole; PMBT = PPI + Metronidazole + Bismuth + Tetracycline.*
***Adherent = documented treatment completion (≥80%).*

Table S2. Risk Factors for Treatment Failure and Post-Treatment Complications (N = 850).

**Variable**	**n (%)**
**Risk Factors**	
Compliance	787 (92.6)
Macroscopic Lesions	159 (18.7)
Age (≥ threshold)	70 (8.2)
Smoking	62 (7.3)
Side Effects	19 (2.2)
Endoscopic Diagnosis	17 (2.0)
Alcohol	1 (0.1)
**Complications**	
Chronic Atrophic Gastritis	110 (12.9)
Peptic Ulcer Disease	10 (1.2)
MALT Lymphoma	2 (0.2)
Idiopathic Thrombocytopenic Purpura	1 (0.1)
Gastric Cancer	0 (0.0)
**Metabolic and Cardiovascular Complications**	
Dyslipidemia	28 (3.3)
Ischemic Heart Disease	3 (0.4)
Myocardial Infarction	1 (0.1)
Atherosclerosis	1 (0.1)

Table S3. Association Between Treatment Regimen and Eradication Failure (N = 196).

Regimen	n	Fail	%	OR (95% CI)	p	aOR (95% CI)	p
PAC (ref.)	151	52	34.4	Reference	—	Reference	—
PACM	13	4	30.8	0.90 (0.27–3.01)	0.861	0.92 (0.25–3.43)	0.906
PMC	4	1	25.0	0.81 (0.09–7.08)	0.851	1.63 (0.17–15.47)	0.668
Other	28	9	32.1	0.92 (0.39–2.18)	0.855	0.85 (0.31–2.33)	0.752

Firth's penalized logistic regression. PAC as reference. Covariates: BMI, sex, hypertension, atrophic gastritis, adherence, pregnancy. No regimen was significantly associated with failure.

Table S4. Antibiotic Therapy Duration and Association with Eradication Outcome.

Variable	Value
**Duration Distribution (N=850, PPI excluded)**	
n with data / Missing	379 / 471 (55.4%)
Mean ± SD (days)	18.0 ± 33.5
Median (IQR)	14 (13–14)
**Duration by Outcome (tested, N=57)**	
Failure (n=25): median (IQR)	14 (10–14)
Success (n=32): median (IQR)	14 (14–14)
**Logistic Regression (N=57)**	
Duration per day — OR (95% CI)	0.997 (0.987–1.006), p = 0.491
≤14 vs >14 days — OR (95% CI)	3.220 (0.606–17.112), p = 0.170

Antibiotic duration = max(termination) − min(initiation) across antibiotics only, PPI excluded. No significant association between duration and failure. Only 57/196 tested patients had complete antibiotic data.
